# Neural Electrical Correlates of Subjective Happiness

**DOI:** 10.1002/hbm.70224

**Published:** 2025-05-27

**Authors:** Wataru Sato, Takanori Kochiyama, Shota Uono

**Affiliations:** ^1^ Psychological Process Research Team Guardian Robot Project, RIKEN Kyoto Japan; ^2^ Brain Activity Imaging Center, ATR‐Promotions Kyoto Japan; ^3^ Division of Disability Sciences, Institute of Human Sciences University of Tsukuba Ibaraki Japan

**Keywords:** fractional amplitude of low‐frequency fluctuation (fALFF), gamma‐band oscillation, precuneus, resting‐state magnetoencephalography (MEG), Subjective Happiness Scale

## Abstract

Happiness is a subjective experience that can serve as the ultimate goal for humans. A recent study that employed resting‐state functional magnetic resonance imaging (fMRI) reported that spontaneous fluctuation (fractional amplitude of low‐frequency fluctuation: fALFF) in the precuneus is negatively associated with subjective happiness. However, little is known about the neural electrical correlates of subjective happiness, which can provide direct evidence of neural activity and insights regarding the underlying psychological, cellular, and neurotransmitter mechanisms. Therefore, we measured 400‐channel whole‐head magnetoencephalography (MEG) during resting state in participants whose subjective happiness was evaluated using questionnaires. We conducted source reconstruction analysis utilizing bandpass‐filtered MEG data and analyzed the fALFF of the band‐limited power time series as an index of spontaneous neural fluctuation. Gamma‐band fALFF values in the right precuneus were negatively associated with subjective happiness scores (partial correlation coefficient = −0.56). These findings indicate that subjective happiness has a neural electrical correlate of reduced spontaneous fluctuation of gamma‐band neuronal oscillations in the right precuneus, and that it could be mediated by a reduction in wandering, clinging self‐consciousness through heightened *N*‐methyl‐d‐aspartate‐dependent gamma‐aminobutyric acid‐ergic parvalbumin inhibitory interneuron activity.

## Graphical abstract

We investigated neural electrical correlates of subjective happiness by analyzing resting‐state magnetoencephalography and measures of subjective happiness. Gamma‐band lower spontaneous neural fluctuation values in the right precuneus were associated with higher subjective happiness scores. These findings suggest that gamma‐band activity in the precuneus implements subjective happiness.

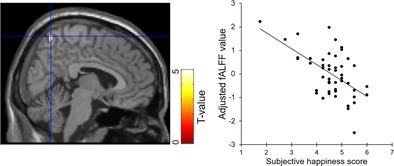

## 1 Introduction

Happiness is a particularly significant subjective experience for humans. A number of ancient and modern philosophers, including Aristotle and Pascal, and contemporary social consensus, as evidenced by the United States Declaration of Independence, proposed that happiness is the ultimate goal of life (Genecov et al. 2024; Kesebir and Diener 2008). Empirical psychological studies of subjective happiness have shown that questionnaires can be used to measure this construct with high reliability and validity (Lyubomirsky and Lepper 1999; Zager Kocjan et al. 2022). Although individuals vary greatly in the sources of their happiness, they generally share a common understanding of what it entails and can accurately assess whether they are happy or unhappy overall (Lyubomirsky and Lepper 1999). Subjective happiness reflects an individual's overall sense of well‐being, encompassing both emotional components—such as a general tendency to experience more positive than negative emotions—and cognitive components, including evaluations of life satisfaction (Lyubomirsky 2001). Moreover, subjective happiness is not merely a consequence of favorable life circumstances but also a contributing factor to various positive outcomes, including better mental and physical health (Lyubomirsky et al. 2005; Paddon and Kampman 2023).

To explore the neural mechanisms underlying subjective happiness, a previous functional magnetic resonance imaging (fMRI) study has investigated resting‐state neural activity associated with measures of subjective happiness (Sato et al. 2019). The researchers calculated the fractional amplitude of low‐frequency fluctuation (fALFF) based on the fMRI signals as an index of spontaneous neural fluctuation (Zang et al. 2007; Zou et al. 2008; for a review, see Canario et al. 2021). The results showed that lower fALFF values in the right precuneus (i.e., the medial aspect of the posterior parietal lobe; Cavanna and Trimble 2006) were associated with higher subjective happiness scores, suggesting that precuneus activity is an objective biomarker of subjective happiness (Figure S1). Furthermore, the results provide insight into the information‐processing mechanisms underlying subjective happiness, based on previous neuroscientific findings on the precuneus. For example, several functional neuroimaging studies in humans have reported that resting‐state blood flow in the precuneus increases during depressive episodes (Cao et al. 2024; Guan et al. 2022; Jing et al. 2013; Smith et al. 2009; Wei et al. 2015; Zhang et al. 2021; for a review, see Li et al. 2017) and decreases after improvements in mental health (Dumas et al. 2012; Guan et al. 2022; Nie et al. 2022; Yang et al. 2019), supporting a psychological continua model of subjective happiness and depression (McGreal and Joseph 1993; Spinhoven et al. 2015, 2021; Stănculescu 2022). Other functional neuroimaging studies demonstrated that stimulus‐evoked precuneus activation is associated with negative self‐referential mental activity (Johnson et al. 2009, 2006; Kim et al. 2020), mind‐wandering (i.e., stimulus‐independent thoughts about the past and future) (Christoff et al. 2009; Mason et al. 2007; Weissman et al. 2006; for a review, see Fox et al. 2015), and clinging, attached experiences (e.g., ruminative thinking and craving) (Burkhouse et al. 2017; Cooney et al. 2010; Dong et al. 2020; Yoon et al. 2023; Zhou et al. 2021; for reviews, see Makovac et al. 2020; Zhou et al. 2020), all of which are negatively associated with subjective happiness in psychological research (Abbe et al. 2003; Gupta and Agrawal 2022; Killingsworth and Gilbert 2010; Lyubomirsky and Ross 1999; Sahdra et al. 2016; Webb et al. 2021). Thus, the fMRI findings showing a negative association between fALFF values in the right precuneus and subjective happiness could provide objective and unique insights.

However, the neural electrical correlates of subjective happiness remain unidentified. This could be important because fMRI only provides an indirect measure of neural activity, based on the blood oxygen level‐dependent (BOLD) effect. Additionally, electric signal data can further elucidate psychological and/or psychiatric characteristics (for reviews, see Newson and Thiagarajan 2019; Perrottelli et al. 2021; Strafella et al. 2022). Furthermore, neural electrical signals can provide insights into the underlying cellular and neurotransmitter mechanisms. For example, several animal studies have shown that dysfunction in the parvalbumin inhibitory interneurons, their *N*‐methyl‐d‐aspartate (NMDA) receptors, or their gamma‐aminobutyric acid (GABA) release leads to increased spontaneous gamma‐band (> 30 Hz; Adrian 1942) activity (e.g., Cho et al. 2015; Guyon et al. 2021; for a review, see McNally and McCarley 2016). To record neural electrical activity in humans, electrophysiological studies based on electroencephalography (EEG) or magnetoencephalography (MEG) are required (Hari and Puce 2023). Although several previous electrophysiological studies have investigated the association between emotional or cognitive subjective well‐being and neural electrical activity (Alessandri et al. 2015; Cannard et al. 2021; Hagemann et al. 1999; Hall and Petruzzello 1999; Isbel et al. 2019; Jacobs and Snyder 1996; Papousek et al. 2019; Shankman et al. 2011, 2005; Sutton and Davidson 1997; Tomarken et al. 1992; Urry et al. 2004; Xu et al. 2018; for reviews, see de Vries et al. 2023; Richter et al. 2024; Table S1), few studies have examined subjective happiness. An exceptional study assessed subjective happiness but found no significant association between subjective happiness and frontal alpha asymmetry on EEG (Day et al. 2019). Several other studies provided indirect evidence regarding this issue. First, several studies in monkeys and humans reported that fMRI signals in response to stimuli correspond to gamma‐band activity (Conner et al. 2011; Engell et al. 2012; Lachaux et al. 2007; Logothetis et al. 2001; Mukamel et al. 2005). Second, several previous electrophysiological studies on monkeys (Leopold et al. 2003) and humans (Foster et al. 2015; Keller et al. 2013; Liu et al. 2010; Nir et al. 2008) suggested that low‐frequency fluctuations of gamma‐band activity during rest show patterns identical to spontaneous fluctuations of fMRI signals. Third, as mentioned previously, a resting‐state fMRI study showed a negative association between spontaneous fluctuation of fMRI signals in the precuneus and subjective happiness scores (Sato et al. 2019). Based on these findings, we hypothesized that spontaneous fluctuation of gamma‐band activity in the right precuneus might be negatively associated with subjective happiness scores.

To test this hypothesis, this study investigated the neural electrical correlates of subjective happiness. We measured 400‐channel whole‐head MEG during resting state in participants and assessed their level of subjective happiness using questionnaires (Lyubomirsky and Lepper 1999). MEG can record electric neural activity noninvasively from healthy participants (Hari and Puce 2023) and has better spatial resolution than EEG (Fred et al. 2022; Hedrich et al. 2017), particularly when high‐density channels are used (Wens 2023). MEG data were bandpass‐filtered into theta (4–8 Hz), alpha (8–12 Hz), beta (12–30 Hz), and gamma (30–60 Hz) frequency bands. Source reconstruction was performed using the empirical Bayesian beamformer algorithm (Belardinelli et al. 2012), which has demonstrated strong performance in resting‐state analyses (Little et al. 2018). To quantify spontaneous neural activity, we calculated the fALFF, defined as the ratio of Fourier amplitudes within a specific low‐frequency range (0.01–0.1 Hz) to those across the entire frequency range (Yan et al. 2016), for the band‐limited power time series in each voxel of the source‐reconstructed images. A voxel‐wise multiple regression analysis was conducted, with fALFF values as the dependent variable and subjective happiness scores as the independent variable. Sex, age, and full‐scale intelligence quotient (IQ) were included as covariates of no interest. We predicted that subjective happiness scores would be negatively associated with the fALFF values of gamma‐band activity in the right precuneus.

## 2 Methods

### 2.1 Participants

The study included 51 volunteers (26 women and 25 men; mean ± SD age, 22.3 ± 4.4 years for the entire sample, 22.0 ± 5.6 years for women, and 22.6 ± 3.3 years for men; there was no significant sex difference, *t*(46) = 0.41, *p* = 0.684). Exclusion criteria included inability to complete questionnaires, inability to undergo MRI, and psychiatric disorders. To confirm the latter criterion, the participants underwent the Mini‐International Neuropsychiatric Interview (Sheehan et al. 1998), a short structured diagnostic interview, by a psychologist. The interview did not identify any neuropsychiatric conditions among the participants. The participants were right‐handed, as assessed by the Edinburgh Handedness Inventory (Oldfield 1971). Full‐scale IQs were also measured using the WAIS‐III (Nihon Bunka Kagakusha, Tokyo, Japan), with all participants showing scores within the normal range (mean ± SD = 121.7 ± 8.6 for the entire sample, 122.2 ± 8.2 for women, 121.7 ± 9.0 for men; no significant sex difference, *t*(46) = 0.37, *p* = 0.713). The participants were explained the experimental procedure and provided informed consent. The study was approved by the Ethics Committee of the Primate Research Institute, Kyoto University, Japan, and conducted in accordance with the institutional ethical provisions and Declaration of Helsinki.

### 2.2 Psychological Questionnaires

The Japanese version of the Subjective Happiness Scale (Lyubomirsky and Lepper 1999; Shimai et al. 2004), a four‐item measure of global subjective happiness, was used to measure the subjective happiness of participants. Each item ranged from 1 to 7, with higher scores reflecting greater happiness, and a single composite score for global subjective happiness was calculated by averaging responses to the four items. The reliability and validity of the questionnaire have been verified in Japanese participants (Shimai et al. 2004). The present study was part of a larger project investigating personalities and mental health, financially supported by the Japan Society for the Promotion of Science Funding Program for Next Generation World‐Leading Researchers (LZ008). The results for the association between brain structures or functions and psychological characteristics have been reported elsewhere (Kubota et al. 2019, 2016; Sato et al. 2016, 2015, 2019, 2020; Uono et al. 2017; Yoshimura et al. 2017).

### 2.3 Procedure

The participants completed an eyes‐open resting‐state task lasting 3 min. A small white fixation cross on a black background was continuously presented at the center of the screen. The participants were instructed to fixate on the cross and relax without thinking of any specific contents. We acquired data only under the eyes‐open condition to reduce the drowsiness/sleep promotion effect associated with eye closure (Putilov 2016). A previous resting‐state MEG study has reported that drowsiness was present in 55% of data acquired in the closed‐eyes resting state despite the instruction to stay awake, and the change from alert to drowsy states influenced the spectral power of neural activity (Strijbis et al. 2022). While the relative advantages of the resting state EEG/MEG activity between open‐ and closed‐eyes conditions in test–retest reliability remain uncertain (Duan et al. 2021), a previous study reported that the reliability of the eyes‐open, compared with the eyes‐closed, condition was higher at the source level (Ding et al. 2022). We acquired data within 3 min based on the evidence‐based minimum recommended duration for recording good resting‐state MEG gamma activity (Wiesman et al. 2022).

### 2.4 MEG Acquisition

MEG data were obtained in an electromagnetically shielded room using a 400‐channel (210‐channel axial and 190‐channel planar gradiometers), whole‐head supine position system (PQ1400RM; Yokogawa Electric Corp., Kanagawa, Japan). MEG systems typically employ superconducting quantum interference device (SQUID) sensors to detect the extremely weak magnetic fields generated by neuronal electrical activity (Cohen 1972; Kim and Davis 2021). The system used in this study featured high‐sensitivity SQUID sensors with a magnetic field resolution of $3\;\text{fT}/\sqrt{\mathrm{Hz}}$ (Shimogawara et al. 2004). A forehead strap was used to stabilize the head position. MEG data were sampled at 1000 Hz through a bandpass filter of 0.05–200 Hz. An example of the recorded MEG signals is provided in Figure S2. Vertical and horizontal electrooculograms (EOGs) were recorded simultaneously.

To determine the head position within the MEG sensor system, five Revie head position indicator coils were mounted on the participants' scalp. Electromagnetic calibration of the coil positions was conducted before and after each MEG recording session. The head shape and calibration coil positions were digitized using a three‐dimensional laser‐optical scanner and a stylus marker (FastSCAN Cobra, Polhemus Inc., Colchester, VT, USA), and were used to coregister the MEG sensor locations to the anatomical space defined by the MRI.

### 2.5 Anatomical MRI Acquisition

Anatomical MRI was performed with a 3‐T scanning system (Magnetom Trio A, Tim System; Siemens Medical Solutions, Malvern, PA, USA) at the ATR Brain Activity Imaging Center using a 12‐channel head coil. Small elastic pads were placed on both sides of the head to minimize head motion. A T1‐weighted high‐resolution anatomical image was obtained using a magnetization‐prepared rapid‐acquisition gradient‐echo sequence (repetition time = 2250 ms; echo time = 3.06 ms; flip angle = 9°; inversion time = 1000 ms; GRAPPA acceleration factor = 2; 208 sagittal slices; slice thickness = 1 mm; field of view = 256 × 256 mm; voxel size = 1 × 1 × 1 mm). MEG and MRI were conducted on different days within a period of 1.5 months, and the sequence varied across participants.

### 2.6 MEG Analyses

**FIGURE 1:**
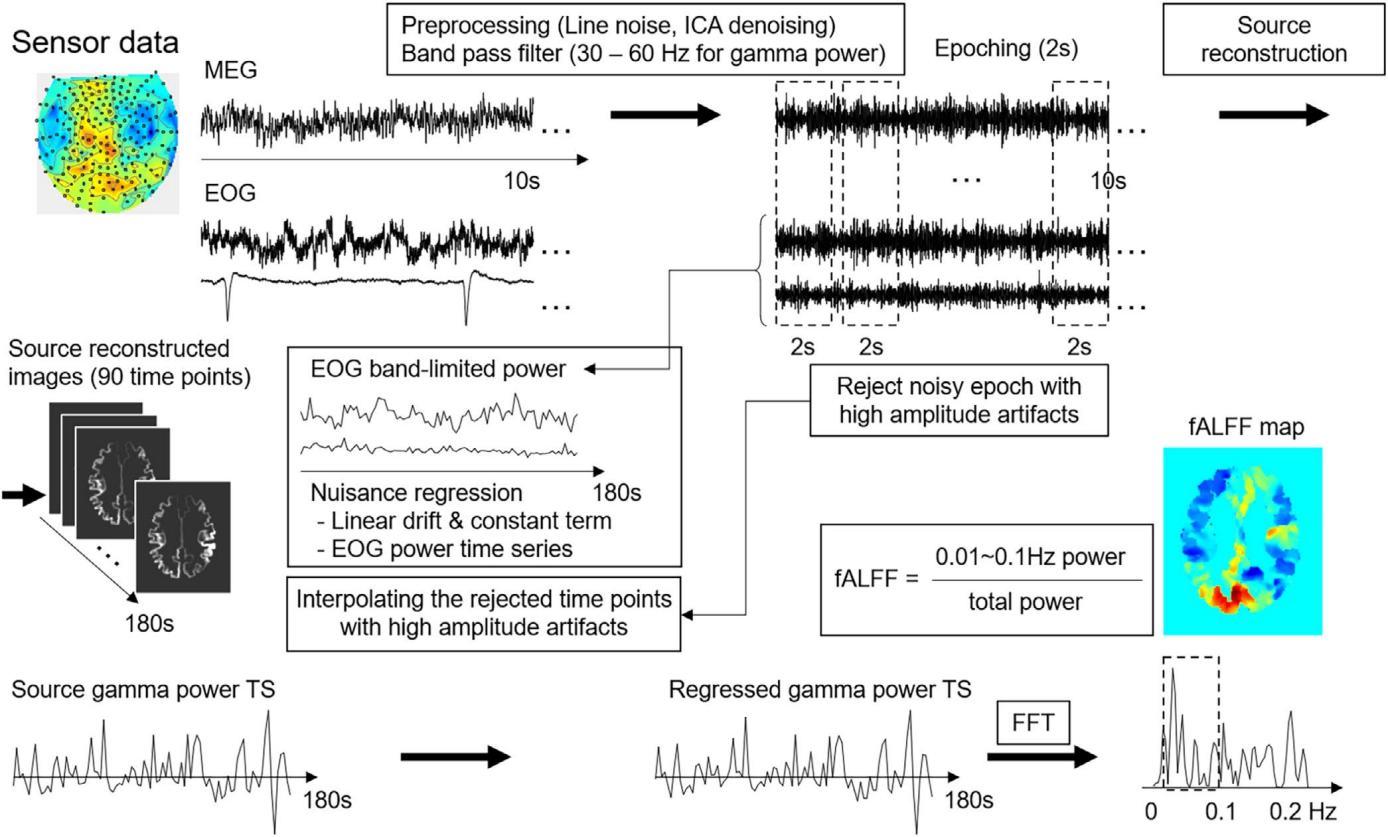
Flowchart of magnetoencephalography (MEG) data analysis. Vertical and horizontal electrooculography (EOG) data were also used. The analysis included preprocessing, which involved line noise filtering, independent component analysis (ICA) denoising, bandpass filtering in four frequency bands (theta [4–8 Hz], alpha [8–12 Hz], beta [12–30 Hz], and gamma [30–60 Hz]), epoching, and rejecting bad epochs; source reconstruction, creating band‐limited power image time series data; and fractional amplitude of low‐frequency fluctuation (fALFF) analysis, regressing out nuisance signals, correcting bad timepoints, and generating the fALFF map for statistical analysis using the Fast Fourier Transform (FFT).

The data were analyzed using the Statistical Parametric Mapping (SPM) 12 package (http://www.fil.ion.ucl.ac.uk/spm) and Fieldtrip software (Oostenveld et al. 2011; https://www.fieldtriptoolbox.org/) implemented in MATLAB R2018a (Mathworks, Natick, MA). Figure 1 presents the data analysis, including preprocessing and fALFF calculation. Figure S2 presents examples of the processed MEG data.

#### 2.6.1 Preprocessing

Continuous MEG data were down‐sampled to 200 Hz (cf. Badura‐Brack et al. 2017); the 60 Hz line noise and its harmonics were removed using a Discrete Fourier transform filter. After removing data from the initial and final periods, data from 180 s were analyzed. The data were subjected to independent component analyses to remove artifacts. The Infomax algorithm implemented in the EEGLAB toolbox (Delorme and Makeig 2004; https://sccn.ucsd.edu/eeglab/index.php) was used. The artifacts were identified using a semiautomatic approach based on various metrics, including the correlation of component time series with EOG signals (eye blink and movement artifacts), kurtosis of the component time series (eye, muscle, and electrical artifacts, cf. Delorme et al. 2007), and the spectral power in frequency bands (cardiac and muscular artifacts), and confirmed by visual inspection of component time courses and topoplots.

The cleaned data were bandpass‐filtered (fifth‐order Butterworth two‐pass filter) into theta (4–8 Hz), alpha (8–12 Hz), beta (12–30 Hz), and gamma (30–60 Hz) bands, and divided into 902,000‐ms epochs. Epochs with high‐amplitude artifacts were marked and excluded from source reconstruction. The global signal (root mean square power of the whole brain signals in each epoch) was used to detect the outlier epochs, that is, with global signal outside five scaled median absolute deviations from the median of all epochs (see MATLAB function outlier.m for additional details).

For MEG source reconstruction, the anatomical MRI of each participant was segmented and spatially normalized to the Montreal Neurological Institute (MNI) space using the unified segmentation‐spatial normalization approach (Ashburner and Friston 2005). Then, inversion of the normalization transformation was used to warp a canonical cortical mesh in the MNI space to the individual cortical mesh (Mattout et al. 2007). The cortical mesh described the source locations according to 8196 vertices (i.e., “normal” size). Next, the MEG sensors were coregistered to the individual anatomical MRI by matching the positions of the three fiducials (nasion and R‐ and L‐preauricular points) and head shape. Then the forward model was computed using the “MEG local spheres” model (Huang et al. 1999) and assuming that the source orientations were constrained to be normal to the cortical mesh.

Following inversion of the forward model, we conducted cortical surface‐based source reconstruction using an Empirical Bayesian beamformer (Belardinelli et al. 2012; Little et al. 2018). Source reconstruction was performed for each frequency band separately. The parameters of the inversion were based on SPM default settings, but did not use the post‐stimulus time (PST) Hanning window option, which is useful for task‐based MEG analysis.

For each participant, we obtained 90 timepoint three‐dimensional source‐reconstructed 3D images in the MNI space, in which each voxel's value was the band‐limited power of resting‐state activity, after converting from a cortical mesh (2D surface) to 3D volume (in voxel coordinates) using nonlinear interpolation (spm_eeg_inv_Mesh2Voxels.m in SPM12 toolbox). Finally, these source‐reconstructed images were spatially smoothed using an 8 mm full‐width isotropic Gaussian kernel at half‐maximum to improve the signal‐to‐noise ratio and compensate for the anatomical variability among participants.

#### 2.6.2 Calculation of fALFF

To measure the regional intrinsic spontaneous fluctuation in the resting state, we computed the fALFF (Zang et al. 2007; Zou et al. 2008) using individual preprocessed band‐limited power image time series. Prior to fALFF computation, we denoised the images and removed the artifacts from the band‐limited power image time series data, similar to the conventional resting‐state fMRI analysis (cf. Caballero‐Gaudes and Reynolds 2017). The outlier volumes from timepoints of the band‐limited power data with high‐amplitude artifacts (corresponding to bad epochs) were censored and interpolated. Spline interpolation was applied to the entire time series. Nuisance regression (i.e., removal of nuisance regressors) was performed to reduce the effect of eye artifacts and other noise on low‐frequency components, where nuisance regressors included a constant term, linear drift term, and two band‐limited power regressors of vertical and horizontal EOG signals. The denoised band‐limited power time series data were converted into the frequency domain using a Fast Fourier Transform. Then the Fourier amplitude was obtained using the MATLAB function y_alff_falff.m in DPABI toolbox (Yan et al. 2016; http://rfmri.org/). The fALFF is the ratio between the sum of Fourier amplitudes within a specific low‐frequency range (0.01–0.1 Hz) and across the entire frequency range. The fALFF calculation was repeated for each voxel in the whole brain to create an fALFF map for each band, which was entered into group‐level analysis. The fALFF value at each voxel was standardized (i.e., *Z*‐score was calculated) to reduce the potential variability of global effects across participants.

#### 2.6.3 Statistical Analysis

Statistical analyses were conducted using SPM12, which can resolve the problem of familywise error (FWE) corrections for multiple voxels with spatial covariances (Kiebel and Friston 2004; Worsley et al. 1996). To evaluate the association between subjective happiness and regional intrinsic brain activities, we conducted a voxel‐wise multiple regression analysis in SPM12 using fALFF value as the dependent variable, subjective happiness score as the independent variable, and sex, age, and full‐scale IQ as covariates of no interest. These covariates were used in a previous study that analyzed the relationship between fMRI fALFF values and subjective happiness (Sato et al. 2019). The relationship between subjective happiness scores and fALFF values was tested using *t*‐statistics and reported as *Z*‐scores by transforming *t*‐value into the standard normal distribution. Voxels were deemed to be statistically significant when the extent *p*‐value was < 0.05 after FWE correction for multiple comparisons at the peak level based on the random field theory (Worsley et al. 1996). Small volume correction (SVC) was conducted for the bilateral precuneus, which was the primary region of interest (ROI). The ROI was anatomically defined using the automated anatomical labeling (AAL) atlas (Tzourio‐Mazoyer et al. 2002). To explore the involvement of other brain regions, we conducted the same voxel‐wise multiple regression analyses for the whole brain. In addition, for descriptive purposes, we conducted SVC using the aforementioned AAL‐based method for the bilateral anterior cingulate cortex and middle frontal gyrus, as previous studies have reported abnormally increased resting‐state activity in these regions in patients with depression (Kong et al. 2017; Zhou et al. 2017). To further investigate the effect of covariates (Hyatt et al. 2020), we conducted the regression analysis for gamma‐band activity with the precuneus ROI without the covariates, and with the covariates of interaction of happiness × sex.

The relationship between subjective happiness score and fALFF value was illustrated by plotting the values extracted from the cluster against the subjective happiness scores after adjusting for effects (i.e., sex, age, and full‐scale IQ).

## 3 Results

### 3.1 Subjective Happiness Ratings

The mean ± SD subjective happiness rating was 4.6 ± 0.8, in good agreement with a previous standardization study (mean ± SD, 4.7 ± 1.1; Shimai et al. 2004). The range was 1.8–6.0 (i.e., 70% of the entire 1–7 range). There were no significant sex differences, although scores were slightly higher for women than for men (mean ± SD, 4.8 ± 0.6 and 4.4 ± 0.9, respectively, *t*(46) = 1.81, *p* = 0.078), in agreement with previous studies reporting no significant sex difference in subjective happiness ratings with larger samples (> 1000; Esteban‐Gonzalo et al. 2020; Extremera and Ferndez‐Berrocal 2014).

### 3.2 Subjective Happiness–fALFF Associations

**FIGURE 2:**
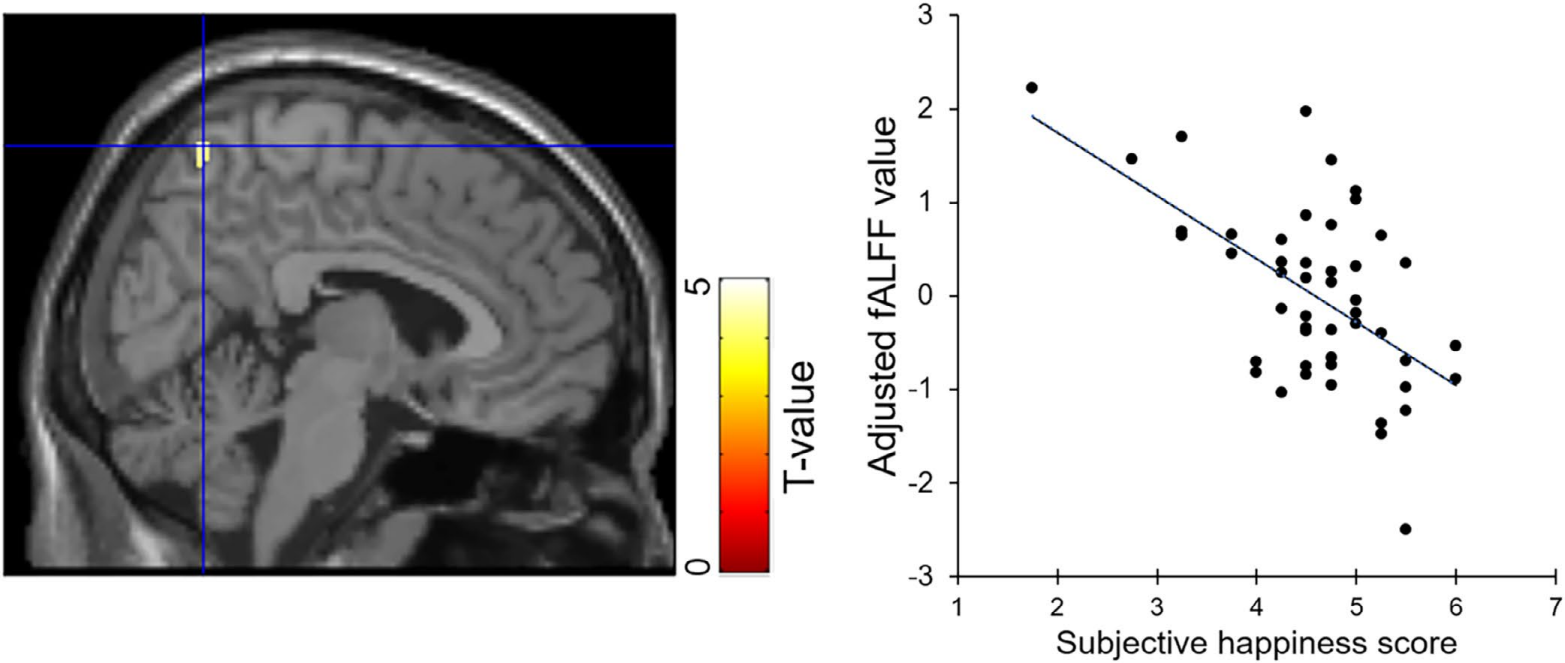
Brain region showing a significant negative association between the subjective happiness score and fractional amplitude of low‐frequency fluctuation (fALFF) value of gamma‐band power amplitude. (Left) A statistical parametric map (*p* < 0.05, peak‐level familywise error [FWE] corrected with a small volume correction [SVC] over the bilateral precuneus) for group analysis of the fALFF map. The area is overlaid on a canonical brain image (single_subj_T1.nii in SPM). The blue cross indicates the location of the peak voxel (*x* = 6, *y* = −62, *z* = 66; *T*_43_ = 4.43, *P*_SVC‐FWE_ = 0.018, cluster size = 23 voxels/184 mm^3^, partial correlation coefficient = −0.56). Red‐white color scale indicates the *T*‐value. (Right) Scatter plot of the adjusted fALFF value of this cluster as a function of the subjective happiness score. Effects of no interest (age, sex, and full‐scale intelligence quotient) were regressed out.

We analyzed the fALFF values for the theta (4–8 Hz), alpha (8–12 Hz), beta (12–30 Hz), and gamma (30–60 Hz) bands using multiple regression analysis with subjective happiness score as the independent variable and sex, age, and full‐scale IQ as covariates. There was a significant negative relationship between subjective happiness score and fALFF value of the gamma‐band activity in the right precuneus (*x* = 6, *y* = −62, *z* = 66; *T*_43_ = 4.43, *P*_SVC‐FWE_ = 0.018, cluster size = 23 voxels/184 mm^3^, partial correlation coefficient = −0.56; Table 1 and Figure 2). The focus was close to one of the foci showing a significant negative relationship between subjective happiness score and fALFF value in a previous fMRI study (*x* = 9, *y* = −72, *z* = 57; Sato et al. 2019). No significant clusters were found in other brain regions, including the left precuneus, bilateral anterior cingulate cortex, and middle frontal gyrus, in any other bands. We additionally conducted regression analyses without covariates and with an interactive covariate (i.e., the interaction between happiness and sex) and confirmed a significant negative association between subjective happiness score and fALFF value of the gamma‐band activity in the right precuneus (*x* = 6, *y* = −62, *z* = 66; *T*_46_ = 4.19, *P*_SVC‐FWE_ = 0.032 for without covariates; *x* = 6, *y* = −62, *z* = 66; *T*_42_ = 4.16, *P*_SVC‐FWE_ = 0.038 for with an interactive covariate). We also conducted the leverage test to assess outliers, but the results remained unchanged despite excluding two outliers (leverage > 0.21) from the regression analysis (*x* = 8, *y* = −62, *z* = 68; *T*_41_ = 4.55, *P*_SVC‐FWE_ = 0.014).

**TABLE 1:** Statistical summary of fractional amplitude of low‐frequency fluctuation (fALFF) values for theta (4–8 Hz), alpha (8–12 Hz), beta (12–30 Hz), and gamma (30–60 Hz) bands at *x* = 6, *y* = −62, and *z* = 66.

	*T*(43)	Partial correlation	*P*_uncorrected_	*P*_SVC‐FWE_	*F*(4,43)	*R* ^2^
Theta	3.28	−0.45	0.001	0.276	3.26	0.233
Alpha	−1.37	0.21	0.912	0.789	1.06	0.089
Beta	−0.58	0.09	0.717	0.971	0.25	0.022
Gamma	4.43	−0.56	< 0.001	0.018	5.02	0.319

Abbreviations: *F*(4,43): *F* statistics for all covariates of interest (subjective happiness score, sex, age, and full‐scale IQ as covariates); *P*_SVC‐FWE_: *p*‐value corrected for multiple comparisons of specified volume of interest (i.e., the precuneus); *P*_uncorrected_: *p*‐value uncorrected for multiple comparisons; *R* ^2^: coefficient of determination; *T*(43): *t*‐statistics for the negative association between subjective happiness scores and fALFF values.

## 4 Discussion

In this study, we investigated the neural electrical correlates of subjective happiness by recording 400‐channel whole‐head MEG during the resting state and assessing subjective happiness levels using the Subjective Happiness Scale (Lyubomirsky and Lepper 1999). We conducted source‐reconstruction analysis on bandpass‐filtered MEG data, dividing it into theta (4–8 Hz), alpha (8–12 Hz), beta (12–30 Hz), and gamma (30–60 Hz) frequency bands. We then evaluated fALFF values, which represent the ratio of Fourier amplitudes within the 0.01–0.1 Hz range to the entire frequency range (Yan et al. 2016). Consistent with our prediction, the fALFF values of gamma‐band power in the right precuneus were negatively associated with subjective happiness scores. This finding is consistent with that of previous fMRI studies that have reported a negative association between the fALFF value of BOLD signals in the right precuneus and subjective happiness scores (Sato et al. 2019). However, the BOLD signals are an indirect measure of neural activity, and the association between neural electrical activity and subjective happiness has not been proven. Our results are in line with the evidence from basic research that low‐frequency fluctuations of gamma‐band activity correspond to spontaneous fluctuations of BOLD (e.g., Leopold et al. 2003). The results are also in agreement with previous studies showing that resting‐state gamma‐band activity in posterior or central areas was increased in individuals with depression (Dai et al. 2022; Wang et al. 2022; Zhou et al. 2018), positively associated with the level of depression (Zhou et al. 2018) and reduced after improvement in depressive symptoms (Kazemi et al. 2016). Depression is considered the opposite end of the continuum of subjective happiness (McGreal and Joseph 1993). However, no prior EEG or MEG studies have identified the neural electrical correlates of subjective happiness (for reviews, see de Vries et al. 2023; Richter et al. 2024). A previous EEG study assessed subjective happiness but found no significant association with EEG activity (Day et al. 2019), a result that contrasts with our findings. This discrepancy may be attributable to methodological differences, as the previous study analyzed sensor‐level signals from frontal lobe electrodes, whereas our study employed source‐reconstructed signals from whole‐brain MEG data. To the best of our knowledge, our MEG study provides the first evidence linking subjective happiness to neural electrical activity, demonstrating that higher subjective happiness scores are associated with reduced spontaneous fluctuations in resting‐state gamma‐band activity in the right precuneus.

Our findings have implications for understanding the information‐processing mechanisms underlying subjective happiness. As noted in Section 1, prior fMRI research has suggested that decreased activity in the precuneus may correlate with reduced negative self‐referential consciousness (Johnson et al. 2009, 2006), diminished mind‐wandering (Fox et al. 2015; Mason et al. 2007; Weissman et al. 2006), and decreased attachment to clinging experiences, such as ruminative thinking and cravings (Burkhouse et al. 2017; Dong et al. 2020). Interestingly, several electrophysiological studies found that gamma‐band activity is associated with these psychological functions in the precuneus or posterior regions. For instance, intracranial EEG studies reported gamma‐band activation in the medial posterior region, including the precuneus, during self‐referential statement processing (Daitch and Parvizi 2018; Dastjerdi et al. 2011, Foster et al. 2015). An EEG study also found that gamma‐band activity in the posterior region was evoked during mind‐wandering states while participants performed a vigilance task (Qin et al. 2011). Additionally, resting‐state gamma‐band activity in the parietal region was shown to be positively correlated with rumination (Wang et al. 2022). Another EEG study observed that hypnosis treatment for nicotine addiction reduced resting‐state gamma‐band activity in the posterior region of smokers (Li et al. 2017). These neuroscientific findings align with psychological studies showing that happier individuals engage less in self‐reflection than unhappy individuals (Abbe et al. 2003; Lyubomirsky and Ross 1999). Furthermore, research found that people who mind‐wander less report greater happiness (Killingsworth and Gilbert 2010; Webb et al. 2021), and those who are less inclined to clinging report higher levels of subjective well‐being (Gupta and Agrawal 2022; Sahdra et al. 2016). Together with these neuroscientific and psychological findings, our results suggest that lower resting‐state gamma‐band activity in the precuneus may reflect reduced self‐referentiality, mind‐wandering, and/or clinging tendencies, which are associated with greater happiness.

Our finding that subjective happiness is inversely related to resting‐state gamma‐band activity also has implications for understanding the cellular mechanisms underlying happiness. Several animal studies have indicated that GABAergic parvalbumin interneurons play a critical role in generating synchronous gamma‐band oscillations via reciprocal connections receiving NMDA‐dependent excitatory input from pyramidal neurons (e.g., Traub et al. 1996; for a review, see Buzsáki and Wang 2012; McNally and McCarley 2016; Meneghetti et al. 2024). Consistent findings from animal studies showed that impairments in parvalbumin interneurons lead to increased spontaneous gamma‐band activity (Carlén et al. 2012; Cho et al. 2015; Del Pino et al. 2013; Guyon et al. 2021; Saunders et al. 2012; Yu et al. 2022), likely through disinhibition of pyramidal neurons (Alaiyed et al. 2019). Parallel findings in humans, such as postmortem and MRI spectroscopy data, demonstrate reduced GABA levels in the brains of individuals with depression (e.g., Sanacora et al. 1999; for reviews, see Fee et al. 2017; Hu et al. 2023), and some postmortem studies report decreased parvalbumin interneuron counts in patients with depression (Knable et al. 2004; Konradi et al. 2011; Rajkowska et al. 2007). Together with these findings, our result suggests that properly modulated NMDA‐dependent GABAergic interneuron activity may contribute to subjective happiness.

Our findings have practical implications. First, it may be possible to use gamma‐band activity in the right precuneus as a biomarker of subjective happiness. Because happiness is considered the ultimate goal of a good life, policymakers are becoming more interested in measuring and improving people's subjective happiness compared to economic success (de Prycker 2010; Frijters and Krekel 2021). However, the subjective measures of happiness have inherent limitations, for example, people can strategically report distorted data if they are motivated (Frey et al. 2014). Resting‐state EEG or MEG data may provide a complementary objective measure of subjective happiness. It may be useful to combine objective assessment of subjective happiness using EEG/MEG with psychological interventions aimed at enhancing subjective happiness. For instance, a prior EEG study has found that practitioners of transcendental meditation, which fosters self‐awareness with reduced conceptual content (Travis et al. 2010) and increases subjective happiness (Gobec and Travis 2018), exhibited decreased gamma‐band activity in the parietal cortex (Travis et al. 2010). Utilizing biofeedback on neural activity could amplify the effects of such meditation practices in enhancing subjective happiness. Objective assessments of subjective happiness using electrophysiological signals may also be valuable for clinical populations. For instance, a study on patients with depressive disorders reported that greater improvements in subjective happiness following treatment were associated with better clinical outcomes, highlighting its relevance for therapeutic interventions (Feliu‐Soler et al. 2021). Similarly, research on patients with schizophrenia found a significant discrepancy between their self‐reported happiness and their psychiatrists' evaluations, suggesting that assessing subjective happiness could contribute to more effective long‐term treatment strategies (Aunjitsakul et al. 2021). Additionally, in individuals with autism spectrum disorder, changes in subjective happiness levels were positively correlated with improvements in objective functioning, including employment, independent living, and social relationships (Scheeren et al. 2022). These findings suggest that assessing and potentially intervening to enhance subjective happiness in clinical populations could be a promising approach for clinical treatments.

Second, our findings suggest that electrical stimulation, modulating gamma‐band activity in the right precuneus, might improve subjective happiness. This aligns with animal studies where gamma‐band electrical stimulation normalized aberrant behaviors in mice with dysfunctional parvalbumin interneurons exhibiting heightened spontaneous gamma‐band activity (Cho et al. 2015). Additionally, human research provided substantial evidence supporting the use of electrical stimulation for depression treatment (e.g., Nie et al. 2022; O'Reardon et al. 2007; for a review, see Mutz et al. 2018), indicating its potential for enhancing happiness.

Finally, our findings suggest that subjective happiness may be influenced pharmacologically by targeting interneuron activity. Numerous studies on animals and humans have reported that ketamine, an NMDA receptor antagonist, increases spontaneous gamma‐band activity in a dose‐dependent manner (e.g., Sanacora et al. 2014; for a review, see Bianciardi and Uhlhaas 2021). Such findings imply that agents upregulating NMDA‐dependent GABAergic interneuron activity in the precuneus could positively modulate subjective happiness. In summary, our data suggest that objective measurements and evidence‐based psychological, electrical, or pharmacological interventions could be used to enhance subjective happiness.

### 4.1 Limitations

This study had several limitations. First, our sample was limited to a healthy population, so the generalizability to clinical populations remains uncertain. As discussed previously, several studies have found increased resting‐state gamma‐band activity in the posterior region among individuals with depression (Dai et al. 2022; Wang et al. 2022; Zhou et al. 2018). A study also found heightened resting‐state gamma‐band activity in the parietal region in individuals with seizures compared to healthy controls (Arikan et al. 2021). These findings suggest that exploring subjective happiness and its association with resting‐state gamma‐band activity in clinical populations is a crucial area for future research.

Second, we recorded electromagnetic signals from the scalp using MEG, which may have missed signals from deeper brain structures, such as the amygdala (Mikuni et al. 1997; Papadelis et al. 2009). Future studies using alternative electrophysiological techniques, like intracranial EEG (Sato et al. 2011), could reveal gamma‐band activity from additional brain regions associated with subjective happiness.

Lastly, as this study used a cross‐sectional design, causal relationships between gamma‐band activity in the precuneus and subjective happiness cannot be inferred. To address this, longitudinal quasi‐experimental studies could test the MEG‐happiness relationship before and after interventions like meditation training, which has been shown to increase subjective happiness (Gobec and Travis 2018). Experimental studies could also induce subjective happiness through activities like writing about positive memories and measure effects on neural activity, although such laboratory inductions have inherent limitations (Ifcher et al. 2021). Future experimental studies are necessary to build evidence regarding the causal relationship between spontaneous gamma‐band activity in the precuneus and subjective happiness.

## 5 Conclusion

Subjective happiness has long been recognized as an essential aspect of human well‐being. Previous fMRI studies have shown that spontaneous activity in the precuneus is negatively correlated with subjective happiness, suggesting a neural basis for happiness. However, the electrical correlates of this relationship remain unexplored (Table S1), leaving a gap in our understanding of the neural activity directly associated with happiness. This knowledge could provide valuable insights into the psychological, cellular, and neurotransmitter mechanisms underpinning happiness. In this study, we investigated this relationship by recording 400‐channel whole‐head MEG during the resting state in healthy participants and assessing their subjective happiness levels using validated questionnaires. We conducted source‐reconstruction analysis on bandpass‐filtered MEG data (theta, alpha, beta, and gamma bands) and analyzed the fALFF of the band‐limited power time series. A voxel‐wise multiple regression analysis, using fALFF as the dependent variable and subjective happiness score as the independent variable, revealed a negative association between subjective happiness and the fALFF value of gamma‐band activity in the right precuneus. These findings suggest that resting‐state spontaneous fluctuations in gamma‐band activity in the right precuneus serve as an electrical neural correlate of subjective happiness. Our findings, along with previous studies, suggest that lower levels of self‐referential thinking, such as mind‐wandering and attachment‐related thoughts, may contribute to subjective happiness. Additionally, the results imply that NMDA‐dependent GABAergic interneuron activity may play a role in subjective happiness. These findings may facilitate the development of objective measurement tools and novel psychological, electrical, or pharmacological interventions aimed at enhancing subjective happiness. However, our study has several limitations, including the restricted generalizability due to testing only healthy participants, the limited spatial resolution of scalp‐recorded MEG, and the inability to establish causality due to its cross‐sectional design. Future research should address these limitations by studying clinical populations, recording intracranial EEG signals, and employing longitudinal quasi‐experimental designs to further explore the electrical neural correlates of subjective happiness.

## Conflicts of Interest

The authors declare no conflicts of interest.

## Supporting information


**Data S1.** Supporting information.

## Data Availability

The data that support the findings of this study are available on request from the corresponding author. The data are not publicly available due to privacy or ethical restrictions.
