# Supplementary material for: Neural Electrical Correlates of Subjective Happiness
Source: Hum Brain Mapp. 2025 May 27;46(8):e70224. doi: 10.1002/hbm.70224 (PMC12107605; doi:10.1002/hbm.70224)
Supplement: Supplementary file 1 — Data S1. Supporting information. [file HBM-46-e70224-s001.doc]

# Supporting Information

Table S1. Overview of research examining resting-state electric neural correlates of subjective well-being.

| **Authors** | **Neural measure** | **Analyzed space** | **Subjective well-being measure** | **Main findings** |
| --- | --- | --- | --- | --- |
| Our study | MEG | Source | Subjective happiness | Decreased spontaneous fluctuation in gamma-band activity in the right precuneus was associated with subjective happiness. |
| Cannard et al. (2021) | EEG | Sensor | Combined subjective well-being | Temporoparietal right vs. left alpha-band decrease (i.e., right activation) was associated with subjective well-being. |
| Day et al. (2019) | EEG | Sensor | Subjective happiness | Frontal alpha-band asymmetry was not associated with subjective happiness. |
| Isbel et al. (2019) | EEG | Sensor | Dispositional positive and negative emotion | Changes in frontal alpha-band asymmetry were not associated with changes in dispositional emotion. |
| Papousek et al. (2019) | EEG | Sensor | Dispositional positive and negative emotion | Frontal right vs. left alpha-band increase (i.e., left activation) was associated with dispositional positive emotion. |
| Xu et al. (2018) | EEG | Sensor | Combined subjective well-being | Changes in frontal right vs. left alpha-band increase (i.e., left activation) were associated with changes in combined subjective well-being. |
| Alessandri et al. (2015) | EEG | Source | Life satisfaction | Right vs. left alpha-band decrease (i.e., right activation) in the middle temporal gyrus, posterior cingulate, and precuneus was associated with life satisfaction. |
| Shankman et al. (2011) | EEG | Sensor | Dispositional positive and negative emotion | Posterior left vs. right alpha-band increase (i.e., right activation) was associated with negative emotion in females with high positive emotion. |
| Shankman et al. (2005) | EEG | Sensor | Dispositional positive and negative emotion | Posterior right vs. left alpha-band increase (i.e., left activation) was associated with decreased positive emotion. |
| Urry et al. (2004) | EEG | Sensor | Dispositional positive emotion; life satisfaction; psychological well-being | Frontal left vs. right alpha-band decrease (i.e., left activation) was associated with positive emotion, life satisfaction, and psychological well-being. |
| Hagemann et al. (1999) | EEG | Sensor | Dispositional positive and negative emotion | Frontal/temporal/central/parietal alpha-band asymmetry was not associated with dispositional positive/negative emotion. |
| Hall and Petruzzello (1999) | EEG | Sensor | Dispositional positive emotion; life satisfaction | Frontal alpha-band asymmetry was associated with positive and negative emotion and life satisfaction, depending on the level of physical activity. |
| Sutton and Davidson (1997) | EEG | Sensor | Dispositional positive and negative emotion | Frontal alpha-band asymmetry was not associated with dispositional positive/negative emotion. |
| Jacobs and Snyder (1996) | EEG | Sensor | Dispositional positive and negative emotion | Frontal right vs. left alpha-band increase (i.e., left activation) was associated with decreased dispositional negative emotion. |
| Tomarken et al. (1992) | EEG | Sensor | Dispositional positive and negative emotion | Anterior temporal right vs. left alpha-band increase (i.e., left activation) was associated with dispositional positive emotion. |
| MEG, magnetoencephalography; EEG, electroencephalography. | | | | |


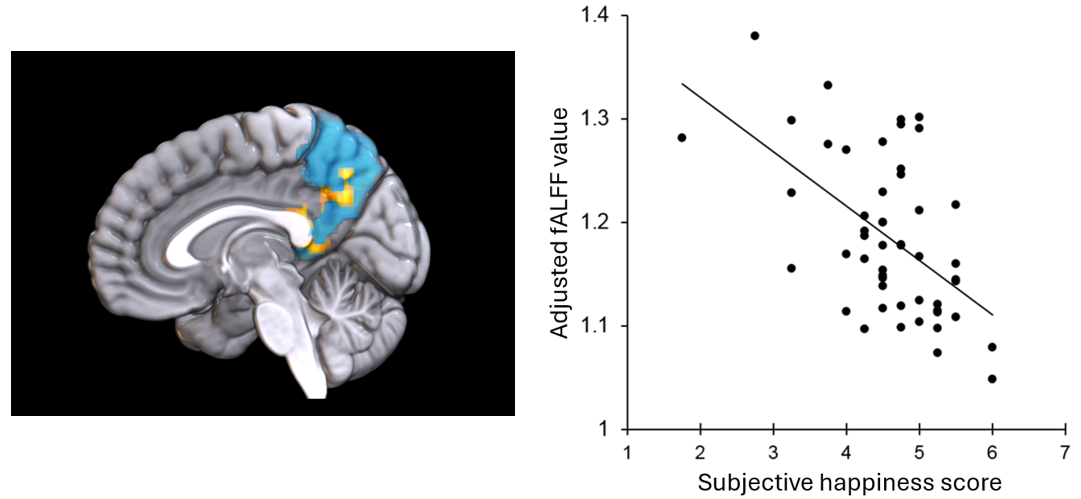


Figure S1. The right precuneus (highlighted in blue on the medial surface of the brain in the left figure) showing a significant negative association between the subjective happiness scores and fractional amplitude of low-frequency fluctuation (fALFF) (highlighted in yellow in the left figure and represented the scatterplot in the right figure) in Sato et al.’s (2019) functional magnetic resonance imaging study.


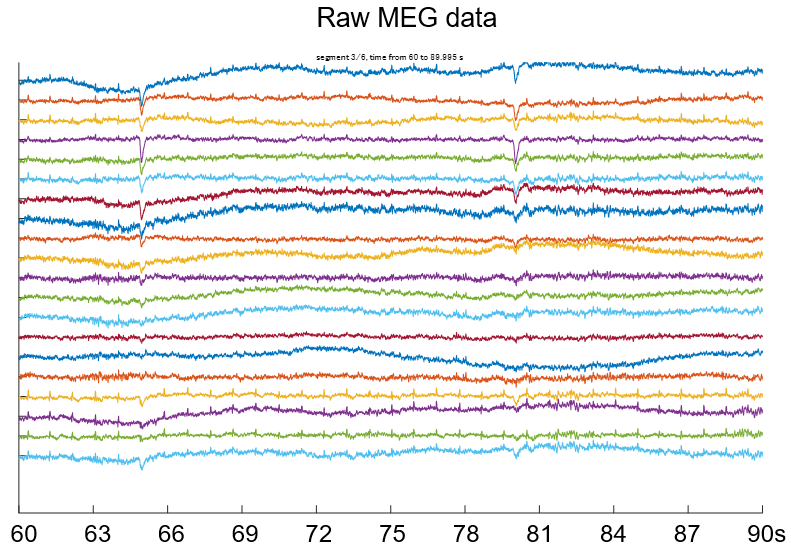


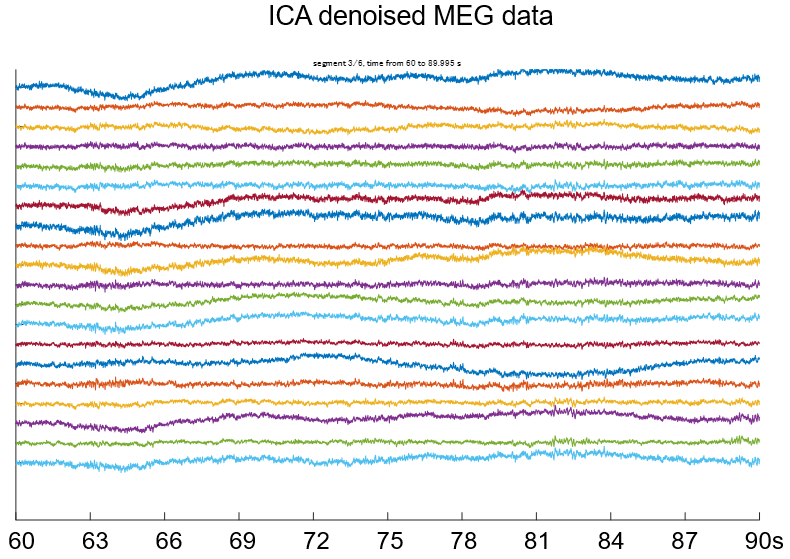


(continued)


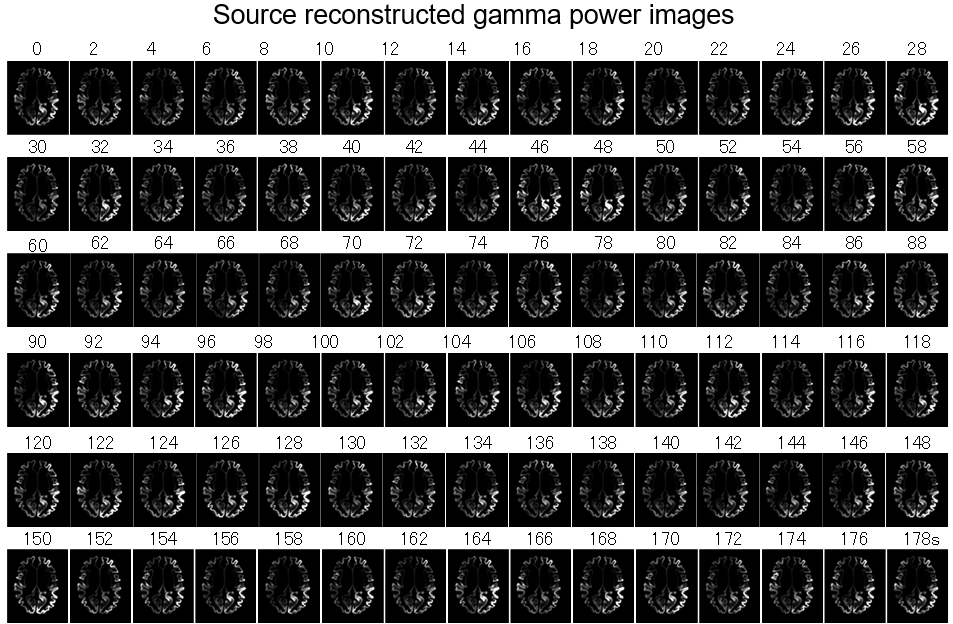


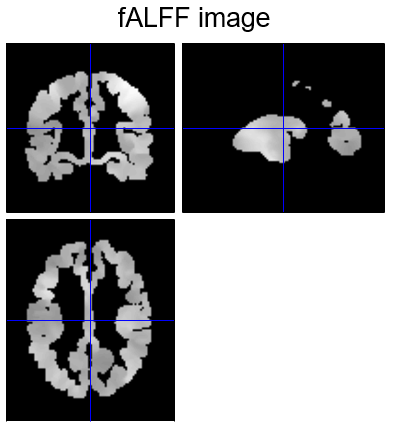


Figure S2. Sample data at each analysis step for a representative subject are presented. The raw magnetoencephalography (MEG) data is shown in the top panel, the independent component analysis (ICA)-denoised data in the second panel, the gamma power image time-series data after source reconstruction (from a specific slice) in the third panel, and the fractional amplitude of low-frequency fluctuation (fALFF) image in the bottom panel.
